# Partnering with healthcare facilities to understand psychosocial distress screening practices among cancer survivors: pilot study implications for study design, recruitment, and data collection

**DOI:** 10.1186/s12913-021-06250-5

**Published:** 2021-03-17

**Authors:** Diane Ng, M. Shayne Gallaway, Grace C. Huang, Theresa Famolaro, Jennifer Boehm, Karen Stachon, Elizabeth A. Rohan

**Affiliations:** 1 Westat, Rockville, MD USA; 2 Centers for Disease Control and Prevention, Division of Cancer Prevention and Control, Atlanta, GA USA; 3 American College of Surgeons, Commission on Cancer, Chicago, IL USA

**Keywords:** Cancer, Electronic health records, Methods, Oncology, Psychological distress, Research design

## Abstract

**Background:**

We sought to understand barriers and facilitators to implementing distress screening (DS) of cancer patients to inform and promote uptake in cancer treatment facilities. We describe the recruitment and data collection challenges and recommendations for assessing DS in oncology treatment facilities.

**Methods:**

We recruited CoC-accredited facilities and collected data from each facility’s electronic health record (EHR). Collected data included cancer diagnosis and demographics, details on DS, and other relevant patient health data. Data were collected by external study staff who were given access to the facility’s EHR system, or by facility staff working locally within their own EHR system. Analyses are based on a pilot study of 9 facilities.

**Results:**

Challenges stemmed from being a multi-facility-based study and local institutional review board (IRB) approval, facility review and approval processes, and issues associated with EHR systems and the lack of DS data standards. Facilities that provided study staff remote-access took longer for recruitment; facilities that performed their own extraction/abstraction took longer to complete data collection.

**Conclusion:**

Examining DS practices and follow-up among cancer survivors necessitated recruiting and working directly with multiple healthcare systems and facilities. There were a number of lessons learned related to recruitment, enrollment, and data collection. Using the facilitators described in this manuscript offers increased potential for working successfully with various cancer centers and insight into partnering with facilities collecting non-standardized DS clinical data.

## Background

Distress in a cancer patient is defined as an unpleasant psychological, social, emotional, and/or spiritual experience that interferes with the ability to effectively cope with a cancer diagnosis, symptoms, or subsequent treatment side-effects [1]. Since 2015, the American College of Surgeons’ Commission on Cancer (CoC) has required distress screening (DS) of cancer patients seen in their accredited facilities [2]. Cancer patients must be screened for distress a minimum of one time at a pivotal medical visit as determined by the program (e.g., diagnosis, beginning and ending treatments, recurrence or progression), and preference should be given to visits at times of greatest risk for distress [2]. Research has demonstrated the downstream beneficial impact on patients, families, cancer outcomes, and the medical system when patient distress is addressed [3, 4].

Successful implementation of routine DS in oncology settings requires thoughtful planning, the feasible and appropriate use of a scientifically valid tool [5, 6], and a host of practical variables within each practice setting (e.g., staffing availability, resource availability, monetary cost of implementation) [6–8]. Despite evidence of the effectiveness of psychosocial services in alleviating distress, many cancer patients who could benefit from these services do not receive them. Barriers exist at multiple levels (i.e., patient, provider, setting) [9, 10].

Prior multi-facility-based healthcare studies have shown that the process for facility recruitment can be lengthy and that many may decline to participate for any number of reasons, such as the lack of staff time, management support, and institutional review board (IRB) issues [11]. While patient recruitment strategies have been well described [12], best practices in studying institutional policies and practices are far less published. Whicher et al. [13] and Anderson et al. [14] describe the importance of identifying and engaging with key leaders within organizations. Successful recruitment of a sufficient number and representative mix of healthcare facilities requires considerable preparation, planning, and flexibility. It is also important to consider that the processes may take longer and be more complex than anticipated from the outset [15]. Among interested healthcare facilities, barriers to participation may still exist, including a lack of financial resources or insufficient staff to carry out requirements necessary to participate in the study [15].

We sought to understand barriers and facilitators to implementing DS to inform and promote uptake in all facilities treating cancer patients. We designed a mixed-method study that included a quantitative review of existing electronic health records (EHRs) and qualitative interviews with health care practitioners. Based on recent recommendations to facilitate the integration of DS and management research into practice [7, 16], here we describe the challenges and facilitators associated with the study design, recruitment, and quantitative data collection from multiple oncology treatment facilities.

## Methods

### Design

The Centers of Disease Control and Prevention (CDC) sponsored this study and contracted with Westat, a research company, to assist with the design and implementation. The study design included specific data items that were collected from EHRs. We solicited feedback on the study design and study materials from social workers who represented medical facilities that were part of a similar DS study [17]. Through a partnership with the CoC, we offered potential facilities credit towards CoC’s *Standard 4.7 Study of Quality* (2016 edition) [2] or for the number of patients accrued for *Standard 9.1 Clinical Research Accrual Study* (2020 Edition) [18] as a direct benefit for their participation in the study.

Study recruitment began in October 2017, with data collection occurring subsequently over 2 years. This study was reviewed and approved by the CDC Human Subjects Review Board (protocol #7225.0), Westat’s Human Subjects Review Board (protocol #6282.07), and the U.S. Office of Management and Budget (OMB Control# 0920–1270). All participating facilities also completed their own institutional review board (IRB) review and approval. The requirement for patient consent was waived in accordance with federal regulations per IRB approvals at each facility, and all methods were carried out in accordance with relevant guidelines and regulations.

### Study sample and recruitment

We focused our study on lung and ovarian cancer patients because of the high potential for these cancers to be associated with distress; lung cancer is the deadliest cancer in the United States [19] and ovarian cancer frequently recurs within a short period of time and has moderate survival [20].

We used data from the American Hospital Association (AHA) Annual Survey 2015 Database [21] to identify facilities of interest. Initially, we had targeted states with high incidence rates of lung and/or ovarian cancer to ensure we had sufficient numbers of cancer survivors represented in the study, particularly for ovarian cancer, which has a lower incidence [19]. We included both CoC and non-CoC-accredited facilities, because we were interested in investigating whether there were differences in DS by accreditation status. Only facilities that reported offering cancer services and having fully implemented EHRs were eligible for inclusion. The initial sampling frame was then stratified into four facility types based on CoC-accreditation status (CoC and non-CoC-accredited) and geographic urbanicity (urban and rural) to achieve a mix of facilities for recruitment. Among the CoC-accredited facilities, we considered cancer program category type as assigned by the CoC (e.g., Comprehensive Community Cancer Program, NCI-Designated Network Cancer Program) during recruitment, with the intention of obtaining facilities across different categories.

We obtained contact information for Cancer Program Managers and Hospital Registrars for a sample of 100 facilities directly from the CoC. We chose to contact these facility staff based on CoC’s suggestion as staff who would understand the current study and are most often involved in directing the flow of information to the cancer committee and decision makers pertaining to DS. Invitation emails were sent to the 100 facilities in three batches on a rolling basis.

For non-accredited facilities, we reached out to Directors of Oncology Services, Directors of Quality Improvement, Directors of Social Services, and Directors of Patient Care/Nursing using contact information obtained from SK&A (https://www.skainfo.com/). SK&A, now OneKey™ by IQVIA, holds a national database of healthcare providers, which is updated on a continuous basis through government and non-government industry sources. We selected these facility staff based on available options from the OneKey™ database who would likely understand and be interested in the current study. We sent information about the current study and an invitation to participate to the identified healthcare facility contacts via email. We offered potential study sites facility-specific feedback reports based on the results of the study as a benefit for participation, and offered CoC-accredited healthcare facilities the additional benefit of receiving credit towards one of two pre-approved CoC standards. Due to a lack of response from non-CoC-accredited facilities, we focused the study scope to CoC-accredited facilities with a minimum number of lung and ovarian cancer cases (< 130 combined) and expanded recruitment to all states. We also promoted the study through professional networks and organizations that would have an interest in DS.

### Study enrollment

Facilities interested in participating in the study underwent a multi-part enrollment process. We held orientation calls with healthcare facility contacts from each facility that expressed interest. The facility staff we engaged with typically included oncology/cancer registry data managers, oncology social workers, and clinical nurses. We provided facilities with an introduction to the study, including information about data collection methods, data security, requirements for IRB submission at the facility, requirements for business associates/data use agreements, a proposed timeline for the study, and benefits of study participation. During this orientation call, we also collected facility-level data on lung and ovarian cancer caseload, each facility’s DS protocol, and information about their EHR system. To aid facility staff in preparing their IRB applications for review at their respective facilities, we provided a template pre-populated with study-specific information.

**Fig. 1:**
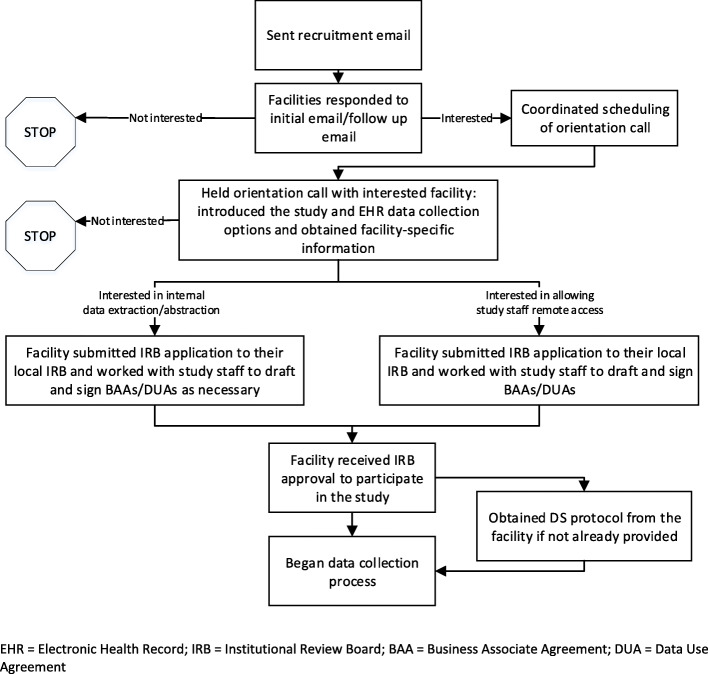
Recruitment and enrollment process

We tracked communication and the status of facilities and followed up periodically, often by phone, to encourage their progression through the enrollment process. After a facility obtained IRB approval and received a Business Associates Agreement (BAA) or Data Use Agreement (DUA) as necessary, we initiated the process of data collection with that facility (see Fig. 1 for a flow diagram of the full recruitment and enrollment process).

During the first enrollment call, we offered two options for facilities to supply their EHR data: 1) study staff (external to the facilities) to remotely access the facility’s EHR system and directly abstract relevant data from patient records, or 2) facility staff to conduct the extraction and abstraction of relevant data from their EHR and submit the data to the study team through a secure file transfer protocol. For healthcare facilities using remote access (option 1) for data collection, the study team signed the healthcare facility BAA or DUA to protect privacy and confidentiality of patient information viewed during the abstraction process.

### Data collection

**Fig. 2:**
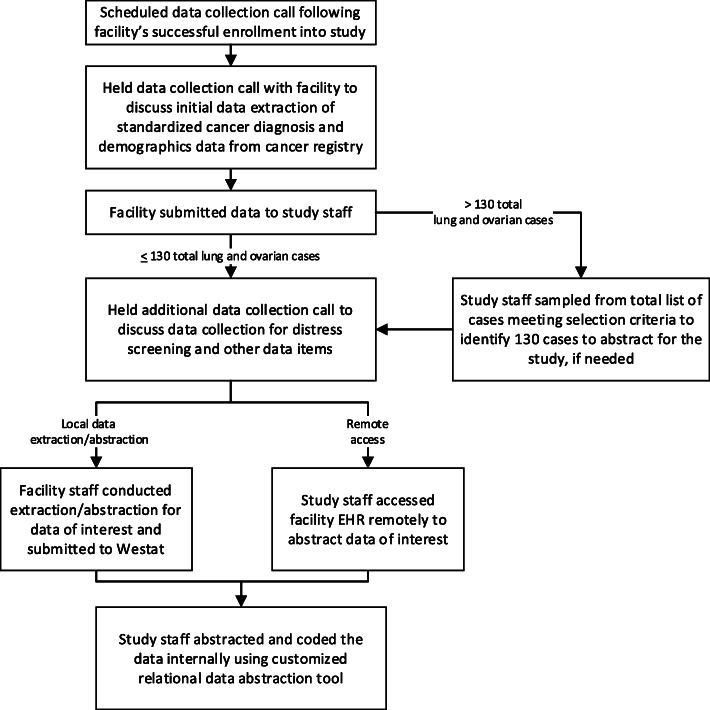
Phases of quantitative data collection processes

Data were requested in two phases. Phase 1: an extraction of standardized data from the facility’s cancer registry database, and Phase 2: a manual abstraction of non-standardized data fields (Fig. 2).

In Phase 1, we requested a case listing of the standardized patient cancer diagnosis and demographic data for all cancer cases that met the following criteria:

- Primary site (International Classification of Disease, Tenth Revision, Clinical Modification (ICD-10-CM) [22]: Lung (C34) or Ovarian (C56)
- Diagnosed in 2016 or 2017
- Diagnosed or treated at the participating facility

Based on a power analysis, we aimed to collect data on a minimum of 2000 patients across all participating facilities. We requested 130 cases from each facility to obtain enough patient data to represent each facility’s DS practices. For facilities with more than 130 cases that met the selection criteria, we statistically sampled 130 cases by cancer type and race/ethnicity strata using data from the cancer diagnosis and demographics case listing.

Phase 2 of data collection involved manual abstraction of information about DS and other relevant patient health information such as healthcare utilization, cigarette smoking, and mental health status. We collaborated with facilities to abstract the DS and these other patient-level data elements for the sample of cases for each facility. During this stage of data collection, we presented facilities with multiple options because of anticipated variation in resources and capacity across facilities. As noted, the two data collection options were for study staff to remotely access and abstract relevant data from patient records through the facility’s EHR system, or for facility staff to extract/abstract data locally and securely transfer a dataset for study staff to conduct a second round of abstraction to maintain consistency in methods across facilities. For facilities that provided remote access into their EHR system, we held at least one training call with facility staff to learn how best to navigate the EHR in order to locate each patient’s DS and the other patient-level data. For facilities that performed their own extraction/abstraction, we held at least one training call with facility staff to review the data elements of interest, the data dictionary and study specifications, and to provide guidance to the facility on how best to extract/abstract the data. After receiving these data, we abstracted and coded the data directly into the study database. For both data abstraction options (remote access and data transfer), we used a customized relational data abstraction tool to perform the abstraction and coding. This tool enabled capture and compilation of a relational database that captured data at the patient-level, information on multiple DS events per each patient, and the subsequent interventions/services received per each DS encounter.

### Analyses

Analyses are based on a pilot study of 9 facilities; data were collected through March 2020 for the pilot study. Six of the 9 programs were recruited via our larger study invitation emails. Three additional facilities were recruited outside of the invitation emails and via professional networks. Data from these 9 facilities were included in the final sample. Additional facilities interested in participating in the study were excluded due to ineligibility (e.g., low case counts, no DS protocol or DS data from period of interest, data not accessible due to transitions in EHR system).

## Results

Of the facilities enrolled into the pilot study, the majority were Comprehensive Community Cancer Programs (56%), urban (89%), and located in the Northeast region of the United States (33%) (Table 1).

**Table 1:** Characteristics of healthcare facilities recruited for the pilot study (*n* = 9)

Characteristics (***n*** = 9)	n	%
Commission on Cancer (CoC) Program Category		
Academic Comprehensive Cancer Program	1	11%
Community Cancer Program	1	11%
Comprehensive Community Cancer Program	5	56%
Integrated Network Cancer Program	2	22%
Urbanicity^a^		
Rural	1	11%
Urban	8	89%
Geographic region		
Midwest	2	22%
Northeast	3	33%
South	2	22%
West	2	22%

^a^Geographic regions were defined using U.S. Census Regions designations. Urbanicity is defined by Core Based Statistical Area (CBSA) type, where healthcare facilities with a CBSA type of “Rural” or “Micropolitan” were classified as Rural and healthcare facilities with a CBSA type of “Metropolitan” were classified as Urban

After successful study recruitment, facilities took an average of 33 days to provide the data extract for Phase 1 of data collection and an average of 136 days to provide the data for Phase 2 (Table 2). Facilities that opted to provide the study staff remote access (EHR access option 1; *n* = 6) took longer for recruitment at an average of 104 days than facilities that performed their own extraction/abstraction (EHR access option 2; *n* = 3) at an average of 77 days, but completed data collection more quickly (127 days versus 253 days, respectively). In terms of documentation of DS in the EHR system, all participating facilities reported having specific and fully integrated fields for DS data. However, DS-related data (particularly follow up data related to DS assessment and referrals following DS) were generally found in open-ended text fields (e.g., physician/staff notes), and four of the nine facilities reported these data in scanned notes or forms.

**Table 2:** Timing of recruitment and data collection by data collection type

Phase of Study	Remote Access (***n*** = 6)			Facility Extraction/Abstraction (***n*** = 3)			Overall (***n*** = 9)		
	Mean (days)	Median (days)	Range (days)	Mean (days)	Median (days)	Range (days)	Mean (days)	Median (days)	Range (days)
**Recruitment:** Recruitment email to IRB/BAA received	104	81	51–247	77	77	73–81	95	77	51–247
**Data collection:** IRB/BAA received to quantitative data collection complete	127	146	42–188	253	197	183–378	169	170	42–378
IRB/BAA received to Phase 1^a^ data collection complete	28	17	6–89	43	38	23–67	33	23	6–89
Phase 2^b^ and all quantitative data collection complete	100	101	36–167	210	160	159–311	136	145	36–311
Both recruitment and data collection	231	223	129–401	330	278	260–451	264	260	129–451

^a^Phase 1: extraction from cancer registry (cancer diagnosis and demographics data)

^b^Phase 2: medical record abstraction (DS and other relevant patient health data)

*IRB* Institutional Review Board, *BAA* Business Associate Agreement

## Discussion

The current study provided several lessons learned related to recruitment, enrollment, and data collection. Some particular challenges associated with this study involved the need to engage healthcare facilities with multiple review and approval processes, identifying the most appropriate partners within the facility, meeting all data collection restrictions, and maximizing flexibility to account for the non-standardization between facilities. A thorough description of the implications associated with study design, recruitment, enrollment, and data collection from our study may help to further expand and integrate similar study methods, designs, and outcomes in implementation science among others executing studies in similar settings.

### Recruitment and enrollment

#### Motivate facilities to participate

Facilities require resources to participate in studies, and often encounter time, money, and staffing limitations [11]. We felt it was important to provide some direct benefits for facility staff efforts and the use of non-monetary benefits helped to successfully achieve the desired study sample. The current study offered direct benefits for participating, including credit towards one of two pre-approved CoC standards and an individualized summary report at the conclusion of the study. The CoC credit was an important factor in motivating CoC-accredited facilities to participate. However, non-CoC-accredited healthcare facilities did not respond to requests to participate in the study, and thus, ultimately, we decided to halt recruitment targeting this group of facilities. It’s not entirely clear why there was a lack of response or capability to participate among non-CoC facilities; however, CoC-accredited hospitals are typically larger teaching hospitals with higher volume and additional services and specialists than non-CoC-accredited hospitals [23]. As well, despite targeting rural facilities in the sampling frame, recruitment of rural facilities was less successful. This may also be related to factors associated with the availability of resources within rural facilities [24].

#### Identify the right facility gatekeeper and project champion

Recruitment required sending a study invitation to a specific staff person, or gatekeeper, as the first entry into each facility. We obtained contact information from CoC cancer committee lists, which included cancer program managers or hospital registrars who were familiar with accreditation processes and internal evaluation requirements. Collaboration with the CoC was essential in obtaining the contact information for the appropriate staff because contact information for hospital staff are often not widely and publicly available. By describing the study interests and methodology at the outset, the gatekeeper was instrumental in directing us to the right project champion for the duration of the study. Characteristics of an ideal project champion included staff that had sufficient understanding about DS procedures and/or oncology data at the facility, worked closely with the target population (i.e., cancer patients), and recognized the benefits of improved DS and follow-up care for patients. Often, these were oncology social workers who provide assessments and follow-up services to patients identified as distressed and were most knowledgeable about sources of distress and resources available to patients. Project champions who saw participation in this study as an opportunity to improve patient care were more invested in their site’s participation. When social workers were not available, gatekeepers helped to identify another staff person who had a similar investment or interest in DS. It was greatly beneficial to learn about the best person to contact at a facility prior to initiating contact. The champion from each facility served a key role in moving the facility forward in the study, and was a staff member who was engaged, invested, and committed to the project.

#### Allow time for enrollment

Each facility had multiple approval processes for study participation. Multiple levels of facility leadership needed to approve the study, either formally or informally, and submission of a local IRB application was required to access patient-level data being requested. Similar to other multicenter studies, there was substantial variability in local IRB requirements, which added time to the process and delayed implementation of our study [15, 25, 26]. Progress also depended on staff availability to participate on calls and complete tasks for the various local approval processes. It was important to keep the contacts engaged and moving forward in the process through regular outreach, while also being cognizant of facility staff schedules that were unpredictable. This often required multiple attempts to make a connection, and it was essential to develop a tracking system and thorough documentation to stay organized and aware of the progress of each facility throughout the process.

#### Understand the variation in facility capabilities

All facilities were unique. While it may have been easier to limit participation (based on certain characteristics or criteria) for a more focused and streamlined design, variation on key variables of interest was critical to detecting differences and disparities across facilities [27]. Some facilities were more experienced with participating in research studies than others, and our study design made intentional considerations to accommodate the diversity across facilities and make the process as smooth as possible. For instance, to facilitate the IRB submission process, a common barrier to research participation [11, 28], we found that providing an IRB information template for facility staff to adapt, helped them complete their applications in a more timely fashion. Maximizing shared guidance, summary materials, and template documents lessened the burden on facility staff and assisted with efficiently streamlining facility documentation requirements.

### Data collection

Data collection challenges stemmed from the use of EHRs and specific types of DS measures. Large variation in EHR products, how these data are collected, and a lack of data interoperability often lead to challenges in harmonizing clinical data for the purpose of research [29, 30]. This is compounded with newer fields of study (e.g., in our study of DS process and outcomes), because the data are not yet standardized. Furthermore, EHR data that are collected locally for healthcare provider use can be extremely heterogeneous across healthcare facilities [30].

#### Understand what, where, and how data are available

Facilities are unique and differ in how data are organized and collected. In this study, some facilities used a combination of data collection methods (e.g., paper charts and EHR systems), or even used multiple EHR systems within their own organization across different departments. Data in EHRs are generally available in different forms as structured, unstructured, or semi-structured data [31]. In the current study, while some DS data (e.g., DS score, reasons for distress) were available in structured data fields, information about follow-up and services as a result of DS were rarely structured and were often only available in unstructured notes. For a study involving multiple sites, it is beneficial to identify commonalities across facilities to assist with standardizing data collection as much as possible. In this study, we were interested in cancer diagnosis and demographic information that we knew CoC-accredited facility cancer registries standardly collected [32]. We were able to use this common framework to guide facilities in providing these data. However, because DS data standards were not available, it was difficult to anticipate how to request these data. While published literature [1, 6, 16, 17, 33] provided some guidance on data standards and available data and data sources, the variability across facilities rendered it insufficient, necessitating a longer than anticipated protocol development phase. The lack of standardized or structured data on follow-up to DS makes it difficult not only for researchers, but also for facility staff and clinicians to track the needs identified by an assessment following a DS and services used to manage distress. Though individual facilities would benefit from structured follow-up data for internal use, there is a need for standardized EHR data collection across facilities to allow for a more global view of the impact of DS on patient outcomes. Ideally, this would involve a collaborative effort between DS and technology experts to develop DS data standards that can be broadly implemented and integrated within EHR systems. This would be in line with the objectives set through the Health Information Technology for Economic and Clinical Health Act (HITECH), resulting in the “meaningful use” of EHRs to make improvements in care [34].

#### Account for differences in data collection and protocols across facilities

Due to the lack of data and protocol standards for DS, it was necessary to account for and reduce data element differences for analysis and interpretation. Facility DS measures differed in a number of aspects, including, but not limited to, the DS tool used; the pivotal medical visit where DS was administered; and the location, frequency, and documentation of DS encounters. Our study design considered the importance of capturing these differences. We were interested in understanding the current landscape of DS and so did not limit participation based on DS tools or protocols. Therefore, we designed the data dictionary and data abstraction tool to account for potential differences across healthcare facilities, within healthcare facilities, and even among individual patients (i.e., some patients received more than one DS).

#### Determine the best way to collect data for the study

It was our experience that data collection involved a significant use of facility staff and/or study staff time, particularly for data abstraction, as has been the case in other studies [11, 17]. Facility staff involvement was necessary for data collection, however time and resources to participate varied depending on EHR abstraction method (i.e., remote or abstraction by facility staff). When facilities could not allow or have the capability to grant remote access to their EHR, facility staff were integral to data collection because staff would often manually abstract data from unstructured fields of the EHR. Training was required in order to guarantee valid and reliable data. However, a benefit to having facility staff abstract data was that they were the most familiar with how DS data were entered into the EHR and would know how to locate these data. Conversely, for facilities that could allow remote access to their EHR, significant study staff time was required for records review and abstraction. Furthermore, the facility staff had to train and support study staff who were accessing, navigating, and abstracting data directly from the facility EHR. It may be preferable and more efficient to limit facility participation to those that can provide the data in a specific format, and important to consider the balance of time, resources, target population, and study outcomes with recruitment challenges.

## Conclusion

Our effort to examine DS practices and follow-up among cancer survivors necessitated recruiting and working directly with multiple healthcare systems and facilities. Challenges stemmed from coordinating data collection from diverse facilities, local IRB and approval processes, and issues associated with the lack of DS data standards in EHR-based data collection. Our pilot study helped to identify potential gaps in DS protocols and disparities among patients to directly inform improvements to DS process administration. We also highlighted the lack of DS data standards that are needed to enable clinicians to more easily utilize DS data for evaluation of successful implementation and measurement of the impact of DS on patients. Data standards could be implemented into EHRs to streamline DS data collection across facilities in a way that is not currently available. Working with oncology facilities poses unique challenges; however, using the facilitators described here offers increased potential for enrolling and working successfully with various cancer centers and insight into partnering with diverse facilities when collecting non-standardized DS clinical data.

## Data Availability

The datasets generated and analyzed during the current study are not publicly available due to it not being possible to remove identifiers to adequately protect subjects’ privacy while still retaining useful variables, but are available from the corresponding author on reasonable request.
